# Spreading of chronic pain between body regions: Prospective cohort study among health care workers

**DOI:** 10.1002/j.1532-2149.2012.00143.x

**Published:** 2012-03-27

**Authors:** LL Andersen, T Clausen, IG Carneiro, A Holtermann

**Affiliations:** National Research Centre for the Working EnvironmentCopenhagen, Denmark

## Abstract

**Objective:**

To estimate the prognostic value of pain in one body region on the risk for developing chronic pain in other body regions.

**Methods:**

Prospective cohort study among 5052 Danish female health care workers responding to a baseline and follow-up questionnaire in 2005 and 2006, respectively. Using adjusted logistic regression analysis, the risk for developing chronic pain (>30 days last year) at follow-up in the low back (among those without low back pain during the last year at baseline) when experiencing sub-chronic (1–30 days last year) or chronic pain in other body regions (i.e., the neck/shoulders and/or the knees at baseline) was modelled. Similar risks were modelled for developing chronic pain in the neck/shoulders and knees.

**Results:**

Chronic pain in the neck/shoulders (OR 3.14; 95% CI 1.74–5.70) or knees (OR 2.57; 95% CI 1.28–5.16) at baseline increased the risk for developing chronic pain in the low back at follow-up. Likewise, chronic pain in the neck/shoulders (OR 2.39; 95% CI 1.36–4.17) or low back (OR 1.82 95%; CI 1.07–3.09) at baseline increased the risk for developing chronic pain in the knees at follow-up. The risk for developing chronic neck/shoulder pain was not significantly increased when having pain in the low back or knees at baseline.

**Conclusion:**

Among health care workers, chronic musculoskeletal pain in one body region increases the risk for developing chronic pain in other pain-free body regions. Prevention of musculoskeletal disorders among health care workers should focus holistically on the musculoskeletal system.

## 1. Introduction

European countries are facing shortage of health care workers, both now and in the future (Simoens et al., 2005). A high level of long-term sickness absenteeism in the health care services exacerbates this problem (Lund et al., 2007). Musculoskeletal disorders (MSD) are the most common causes of long-term sickness absence (LTSA) and work disability in several occupations (Natvig et al., 2002; Morken et al., 2003; van den Heuvel et al., 2004; Holmberg and Thelin, 2006; Bergstrom et al., 2007; Nyman et al., 2007; Bevan et al., 2009; Kamaleri et al., 2009; Andersen et al., 2011b). Among health care workers, pain in the low back, knees and neck/shoulder increases the risk for future LTSA (Andersen et al., 2011a). Knowledge of prognostic factors for developing musculoskeletal pain among health care workers is needed to effectively target preventative strategies.

Workers with physically strenuous work show a high prevalence of MSD (Andersen et al., 2007) and LTSA (Lund et al., 2006). Among health care workers, the annual prevalence of low back pain ranges from 45% to 77% (Smedley et al., 1995; Karahan et al., 2009). Consequently, preventive strategies for MSD often focus on a specific body region, e.g., the concept of back schools (Heymans et al., 2004). However, compensatory behaviour, as for instance altered lifting patterns due to pain in one body region may lead to elevated biomechanical loadings and development of pain in other body regions. Also, long-term pain can lead to central sensitization in which altered signalling in the central nervous system amplifies overall pain perception (Graven-Nielsen, 2006). Psychological factors may also influence the pain processing as treatments such as cognitive therapy can reduce sensations of pain (Lamb et al., 2010). Cross-sectional studies show positive correlations between pain in different body regions (Andersen et al., 2010). However, no previous prospective cohort studies have investigated whether pain in one body region is a risk factor for developing pain in other currently pain-free body regions.

**What's already known about this topic?**

- Cross-sectional studies on chronic pain states show positive associations between pain in different body regions, but less is known about spreading of pain from one region to another.

**What does this study add?**

- Chronic pain in one body region is a risk factor for developing chronic pain in other body regions.
- The pain spreading phenomenon should be considered when treating chronic pain.

The aim of our prospective cohort study among 5052 health care workers was to estimate the prognostic value of pain in one body region on the risk for developing chronic pain in other body regions. We hypothesized that pain in one body region increases the risk for developing chronic pain (>30 days with pain last year at follow-up) in other currently pain-free body regions (0 days with pain last year at baseline).

## 2. Methods

### 2.1 Study design and population

This prospective cohort study involves health care workers in eldercare in Denmark. The baseline survey started in the winter of 2004, but the main data collection took place in the spring of 2005. Data collection for follow-up was, for the most part, conducted in the autumn of 2006, but continued on to spring 2007. At baseline, we sent questionnaires to 12,744 potential participants who were employed in the 36 municipalities. Of these potential respondents, 9949 (78%) completed the questionnaire (Clausen et al., 2011; Andersen et al., 2011a). One municipality decided to withdraw from the cohort during the follow-up period, which meant that the follow-up population consisted of 9847 employees from 327 individual work places in the eldercare services. Of these respondents, 7864 were eligible for the follow-up questionnaire in 2006, of which 6307 responded (80%). Our study population is composed of those that replied both at baseline and follow-up (*n* = 6307). Further, we excluded male respondents (*n* = 234) and respondents who were not directly engaged in the provision of care services (*n* = 1021). Thus, the target population with both baseline and follow-up questionnaire replies consisted of 5052 women being directly engaged in the provision of health-related care services in the Danish eldercare sector. More specifically, the population consists of the following professional groups: registered nurses (3½ years of education), social and health care assistants (34 months of education), social and health care helpers (14 months of education), ‘other care staff’ (various care-related educations with less than 12 months of education), therapists and activity staff (this group primarily consists of physio- and occupational therapists with 3½ years of education) and uneducated care staff and cleaners.

Three groups of health care workers without pain (0 days during last year) in at least one of the body regions at baseline and to whom there was information on follow-up pain-related questions were defined: (1) the neck/shoulders (*n* = 1448), (2) the low back (*n* = 1537) and (3) the knees (*n* = 3158). These three groups were not mutually exclusive, e.g., a participant without pain in the neck/shoulder and low back would appear in both groups 1 and 2. We estimate the risk for developing chronic pain at follow-up in these currently pain-free body regions as described in the following.

### 2.2 Ethical approval

The study has been notified to and registered by Datatilsynet (the Danish Data Protection Agency). According to Danish law, questionnaire- and register-based studies do not need approval by ethical and scientific committees, nor informed consent.

### 2.3 Predictive variables

Participants replied to the standardized Nordic questionnaire on musculoskeletal pain symptoms in the low back, neck/shoulders and knees (Kuorinka et al., 1987). The questionnaire concerned duration of pain in each respective body region during the last year, with the response categories 0 days, 1–7 days, 8–30 days, 31–90 days, more than 90 days but not everyday, and everyday. Subsequently, for the statistical analyses, we trichotomized pain into 0 days (reference), 1–30 days (sub-chronic pain) and more than 30 days (chronic pain) (Sjogaard et al., 2010; Andersen et al., 2011a), respectively.

### 2.4 Outcome variables

At follow-up, the participants again replied to the standardized Nordic questionnaire on musculoskeletal pain symptoms (Kuorinka et al., 1987). There were three separate outcomes:

1. For those without low back pain (0 days last year) and having pain at baseline in neck/shoulder and/or knees at baseline, we estimated the risk for developing chronic low back pain (>30 days last year) at follow-up.
2. For those without neck/shoulder pain (0 days last year) and having pain at baseline in the low back and/or knees at baseline, we estimated the risk for developing chronic neck/shoulder pain (>30 days last year) at follow-up.
3. For those without knee pain (0 days last year) and having pain at baseline in the low back and/or neck/shoulder at baseline, we estimated the risk for developing chronic knee pain (>30 days last year) at follow-up.

### 2.5 Confounders

Potential confounders included age (continuous variable), body mass index (BMI = weight/ height^2^, where BMI < 25 is normal weight, BMI 25–30 is overweight, and BMI > 30 is obese), seniority (years working as health care worker; continuous variable) smoking status (dichotomous variable depicting smoker/non-smoker). Further, we included leisure physical activity, physical workload and psychosocial work conditions as described in the following.

To determine leisure physical activity, we asked the question ‘Which description most precisely covers your pattern of physical activity at leisure time during the last 12 months?’ with the following possible responses: (1) mainly sedentary or light physical activity for less than 2 h per week (e.g., you read, watch television, go to the cinema); (2) light physical activity for 2–4 h per week (e.g., you go for a walk, light gardening, light physical exercise); (3) light physical activity for more than 4 h per week or vigorous physical exercise for 2–4 h per week (e.g., fast jogging or cycling, heavy gardening, exercise where you are sweating and breathing heavily); (4); vigorous physical exercise for more than 4 h per week or taking part in regular competitive sports several times a week (Saltin and Grimby, 1968; Andersen et al., 2011a).

To determine physical workload, we used the Hollman questionnaire asking about body postures and weight lifted during the working day (Hollmann et al., 1999). In summary, the resulting Hollmann Index (scale 0–62, with 62 representing the highest degree of physical workload) is a measure of physical workload on the lower lumbar spine within longer time frames.

We additionally adjusted for four indicators of psychosocial work conditions from the Copenhagen Psychosocial Questionnaire (Pejtersen et al., 2010; Clausen et al., 2011): emotional demands, role conflicts, influence at work and quality of leadership (normalized on a 0–100 scale according to the test-score manual, with 100 representing the highest degree of the measured dimension of the psychosocial work environment).

### 2.6 Statistics

Using logistic regression analyses, we estimated the odds for developing chronic pain at follow-up in the pain-free body regions at baseline. Odds ratios (OR) and 95% confidence intervals (95% CI) were calculated for the different types of body regions. In model 1, we adjusted the analyses for age, and entered the respective body regions together in the model (e.g., mutual adjustment for knee pain and neck/shoulder pain at baseline for the odds of developing low back pain at follow-up). In model 2, we additionally adjusted for BMI, smoking status, leisure physical activity and physical workload. Finally, in model 3, we additionally adjusted for psychosocial work conditions. Because the participants were clustered in workplaces, observations were not statistically independent (Twisk, 2006). We used PROC GENMOD of SAS version 9.2 (SAS Institute, Cary, NC, USA) for all logistic regression analyses and used the ‘repeated subject’ option to adjust for random effects at the workplace level.

## 3. Results

Table 1 shows descriptive statistics for the main study variables. At baseline among the cohort of 5052 health care workers, 22% (low back), 28% (neck/shoulder) and 12% (knees) had chronic pain (>30 days last year), and 31% (low back), 29% (neck/shoulder) and 65% (knees) were pain-free (0 days last year). More than 90% performed either light or moderate physical activity during leisure. More than 40% were either overweight or obese.

**Table 1:** Descriptive background characteristics of the study population at baseline (*n* = 5052) in percentage of study population (categorical variables) or mean and standard deviations (continuous variables)

	*n*	Mean (SD) or %
Age	5052	46.0 (9.0)
Job seniority	4979	8.6 (7.1)
BMI		
Normal	2842	58.8%
Overweight	1415	29.3%
Obese	577	11.9%
Smoking	1776	35.5%
Physical workload^a^	4917	19.4 (9.7)
Leisure physical activity		
Sedentary	183	3.7%
Light physical activity	2039	41.0%
Moderate physical activity	2535	50.9%
Vigorous physical activity	220	4.4%
Psychosocial work environment^b^		
Emotional demands	5011	46.9 (17.9)
Influence at work	5010	45.6 (20.0)
Role clarity	5011	74.3 (14.2)
Leadership quality	4931	57.2 (21.5)
Low back pain		
No pain	1558	31.2%
Subchronic pain	2327	46.6%
Chronic pain	1113	22.3%
Neck and shoulder pain		
No pain	1470	29.4%
Subchronic pain	2113	42.2%
Chronic pain	1423	28.4%
Knee pain		
No pain	3215	64.5%
Subchronic pain	1166	23.4%
Chronic pain	601	12.1%

a Physical workload was estimated from the Hollmann Index (scale 0–62).

b Emotional demands, role conflicts, influence at work and quality of leadership are continuous variables that are scored from 0 to 100. On each variable, a score of 100 indicates a high level of emotional demands, role conflicts, influence at work and quality of leadership.

Table 2 summarizes the OR estimates for developing chronic pain when experiencing sub-chronic or chronic pain (reference 0 days during last year) in the other respective body regions at baseline. Models 1 to 3 show the OR estimates when adjusting for several confounders. In the first model adjusting for age (model 1), chronic pain in any of the three body regions is a risk factor for developing chronic pain in any of the other respective body regions. In the final model adjusting for age, BMI, smoking, seniority, physical workload, leisure physical activity and psychosocial work environment (model 3), the odds for developing chronic neck/shoulder pain was not significantly increased when having pain at baseline in the low back and knees. However, chronic pain at baseline in the neck/shoulders (OR 3.14; 95% CI 1.74–5.70) and knees (OR 2.57; 95% CI 1.28–5.16) increased the odds for developing chronic pain in the low back at follow-up. Likewise, chronic pain at baseline in the neck/shoulders (OR 2.39; 95% CI 1.36–4.17) and low back (OR 1.82; 95% CI 1.07–3.09) increased the odds for developing chronic pain in the knees at follow-up. In all three models, sub-chronic pain was not a risk factor for developing chronic pain in other body regions.

**Table 2:** Odds ratios (OR) for developing chronic pain in the low back, neck/shoulders and knees among health care workers without pain in these respective body regions at baseline. For each of the three body regions, subchronic and chronic pain in the other two body regions are used as risk factors (reference = 0 days with pain last year)

Condition at baseline	Outcome at follow-up^a^	Risk factor at baseline		*n*	Model 1	Model 2	Model 3
					OR (95% CI)	OR (95% CI)	OR (95% CI)
No low back pain (*n* = 1537)	Chronic low back pain (*n* = 82)	Neck/shoulder	No pain (reference)	822	1	1	1
			Subchronic pain	532	1.14 (0.67–1.92)	1.15 (0.67–1.95)	1.17 (0.67–2.02)
			Chronic pain	195	**3.64 (2.08–6.37)**	**3.67 (2.07–6.52)**	**3.14 (1.74–5.70)**
		Knee	No pain	1241	1	1	1
			Subchronic pain	200	1.43 (0.71**–**2.90)	1.54 (0.74**–**3.21)	1.30 (0.62**–**2.75)
			Chronic pain	101	**3.28 (1.82–5.89)**	**2.86 (1.47–5.56)**	**2.57 (1.28–5.16)**
No neck/shoulder pain (*n* = 1448)	Chronic neck/shoulder pain (*n* = 102)	Low back	No pain	822	1	1	1
			Subchronic pain	478	1.24 (0.76**–**2.03)	1.17 (0.69**–**1.97)	1.18 (0.68**–**2.04)
			Chronic pain	157	**2.08 (1.07–4.04)**	1.92 (0.91**–**4.07)	1.86 (0.89**–**3.88)
		Knee	No pain	1130	1	1	1
			Subchronic pain	212	1.40 (0.77**–**2.53)	1.40 (0.75**–**2.60)	1.15 (0.60**–**2.20)
			Chronic pain	116	**1.96 (1.04–3.70)**	1.71 (0.85**–**3.42)	1.79 (0.88**–**3.63)
No knee pain (*n* = 3158)	Chronic knee pain (*n* = 131)	Neck/shoulder	No pain	1130	1	1	1
			Subchronic pain	1355	1.64 (0.95**–**2.83)	1.60 (0.92**–**2.81)	1.53 (0.87**–**2.69)
			Chronic pain	725	**2.47 (1.46–4.21)**	**2.64 (1.53–4.56)**	**2.39 (1.36–4.17)**
		Low back	No pain	1241	1	1	1
			Subchronic pain	1450	1.04 (0.65**–**1.66)	1.07 (0.65**–**1.76)	1.08 (0.65**–**1.80)
			Chronic pain	505	**1.83 (1.15–2.91)**	**1.77 (1.05–2.96)**	**1.82 (1.07–3.09)**

Model 1: Adjusted for age.

Model 2: Adjusted for age, BMI, smoking, seniority, physical workload (Hollman Index) and leisure physical activity.

Model 3: Adjusted for age, BMI, smoking, seniority, physical workload (Hollman Index), leisure physical activity and psychosocial work environment.

a Among those without pain in this specific region at baseline.

Because knee and low back pain were not significant risk factors for developing neck/shoulder pain in the multi-adjusted model (model 3), we considered the control variables. Regarding the psychosocial control variables, higher emotional demands at baseline increased the odds for developing chronic neck/shoulder pain at follow-up (OR 1.02; 95% CI 1.01–1.04).

## 4. Discussion

Our prospective cohort study following 5052 health care workers shows that chronic musculoskeletal pain in one body region is an independent risk factor for developing chronic musculoskeletal pain in other currently pain-free body regions.

Several occupational risk factors for developing musculoskeletal pain exist, e.g., increased risk for back pain from lifting, bending and twisting of the trunk (Hoogendoorn et al., 1999; Kuiper et al., 1999; Lotters et al., 2003). Among health care workers, an association between psychosocial work environment and upper limb MSD exists (Herin et al., 2011). Our study shows that even after adjusting for physical workload and psychosocial work conditions, chronic pain in one body region is an independent prognostic factor for developing chronic pain in the knees and low back.

Our study cannot reveal the mechanisms behind pain development from one body region to another. However, one potential mechanism may be central sensitization. Chronic pain can be a cause of or can lead to central sensitization in which altered signalling in the central nervous system amplifies overall pain perception (Graven-Nielsen, 2006). Cross-sectional studies in adults with chronic neck/shoulder pain have revealed lower pressure pain thresholds in distant non-painful muscles of the leg, indicating central sensitization (Nielsen et al., 2010; Sjors et al., 2011). Pain in one region of the body from a mechanical or inflammation-induced insult activates local nociceptors, generating increased neuronal excitability in the spinal cord, driving the spinal circuits ultimately leading to central sensitization (Winkelstein, 2004). In our study, chronic pain in one body region may have caused central sensitization, thereby increasing the risk for experiencing pain in other body regions at follow-up.

Another mechanism may be that pain in one body region leads to altered lifting patterns and consequently elevated mechanical loading of other pain-free body regions. For instance, knee pain may cause less knee bending during occupational lifting at the expense of more back bending, thus increasing strain on the back. Likewise, back or neck pain may cause less forward bending of the upper body during occupational lifting at the expense of increased knee bending, thus increasing strain on the knees. Accordingly, attempts to compensate for pain in one body region by altering biomechanical lifting patterns may be an adverse type of pain coping for health care workers. Studies should be performed in other groups not exposed to physical labour, e.g., inactive elderly with joint pain, to investigate whether or not biomechanical factors have any influence on pain spreading. Altogether, several contributing factors may explain our observation of spreading of musculoskeletal pain between body regions.

In the multi-adjusted model, the risk for developing chronic neck/shoulder pain was not increased when experiencing pain in the knees and low back, which may be explained by knee bending or back bending not necessarily increasing the biomechanical loading on the neck/shoulders. By contrast, we observed that higher emotional demands at baseline increased the risk for prospectively developing chronic neck/shoulder pain. Previous research among other occupational groups has linked the development of neck/shoulder pain to prolonged tension of upper trapezius muscle (Larsson et al., 2007). However, due to the large sample size the significant association between emotional demands and development of neck/shoulder pain may be a statistical type I error. Future studies should investigate whether improved psychosocial work environment can reduce neck/shoulder pain among health care workers.

Although not statistically tested in the present study, the OR of Table 2 indicate that a dose-response pattern between duration of pain in one region and development of chronic pain in another region exists. Future analyses should investigate this in more detail, also to determine the existence of a possible threshold of pain duration leading to spreading of pain.

### 4.1 Strengths and limitations

There are both strengths and limitations to our study. These strengths are the large sample size, the longitudinal design and the high initial response percentage to the baseline questionnaire. We excluded male respondents (*n* = 234) and respondents who were not directly engaged in the provision of care services (*n* = 1021). The resulting homogenous group of female health care workers limits bias from socio-economic confounding. Also, adjustment for both physical workload and psychosocial work conditions strengthens the validity of our findings. However, the study population limits the generalizability of our findings to female health care workers. The loss of respondents from baseline to follow-up increases to some extent the risk for response bias. Further, the single question on leisure physical activity does not provide detailed information on intensity and duration of physical activity. Also, our study cannot reveal the mechanisms of pain development. Regardless, future epidemiological studies investigating the association between occupational exposures and development of specific MSD should account for current pain in other body regions. Finally, future studies should include several body regions – e.g., wrists, elbows, shoulder, neck, upper back, lower back, hips, knees and ankles – to investigate in more detail the phenomenon of pain spreading between body regions.

## 5. Conclusion

Among health care workers, chronic musculoskeletal pain in one body region increases the risk for developing chronic pain in other pain-free body regions. Prevention of MSD among health care workers should focus holistically on the musculoskeletal system instead of focusing on a specific body region only.
